# Case 3/2019 - Type IIB Tricuspid Atresia, in Natural Evolution, at 21 Years of Age

**DOI:** 10.5935/abc.20190083

**Published:** 2019-05

**Authors:** Edmar Atik, Alessandra Costa Barreto, Maria Angélica Binotto

**Affiliations:** Instituto do Coração do Hospital das Clínicas da Faculdade de Medicina da Universidade de São Paulo, São Paulo, SP - Brazil

**Keywords:** Heart Defects, Congenital, Tricuspid Atresia, Pulmonary Valve Stenosis, Clinical Evolution/methods

## Clinical data

Patient remained asymptomatic from birth until 16 years of age, when he started to show progressive fatigue at exertion, with the use of anti-congestive medication such as furosemide, enalapril, spironolactone, and carvedilol, in addition to warfarin. The diagnosis of heart disease characterized by heart murmur was attained in the first month of life.

Physical examination: good overall status, eupneic, acyanotic, normal pulses in the 4 limbs. Weight: 63 kg, Height: 171 cm, right upper limb blood pressure: 130/90 mmHg, HR: 63 bpm, Sat O_2_: 89%.

Precordium: apex beat was palpable at the 6^th^ left intercostal space in the anterior axillary line and diffuse, with systolic impulses in the left sternal border. Hyperphonetic heart sounds, with irregular splitting of the second heart sound. Moderate intensity ejection systolic murmur at the left upper sternal border with systolic thrill and holosystolic murmur ++/4 at the lower sternal border and at the tip with diastolic murmur ++/4. The liver was palpable 4 cm from the costal border and lungs were clear.

### Complementary examinations

**Figure 1:**
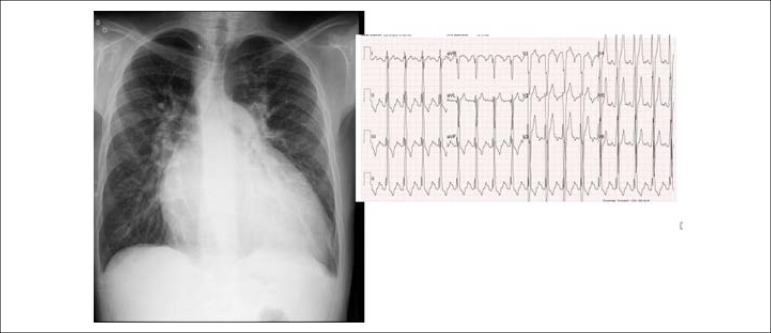
Chest x-ray highlights the marked increase of the cardiac area (CTI = 0.69) with increased pulmonary vascular network in the hila. Electrocardiogram shows left-chamber overload.

**Electrocardiogram:** Sinus rhythm and signs of left-chamber overload, with a narrow QRS of 0.87 ms (AQRS = +110°), a positive T wave in V1 (AT = +10°), and an enlarged P wave in II, III and in F (AP = + 60°) (Figure 1).

**Chest x-ray:** Significantly increased cardiac area on account of the right arch with double contour and left ventricular arch (CTI = 0.69). Increased pulmonary vascular network and bulging middle arch (Figure 1).

**Figure 2:**
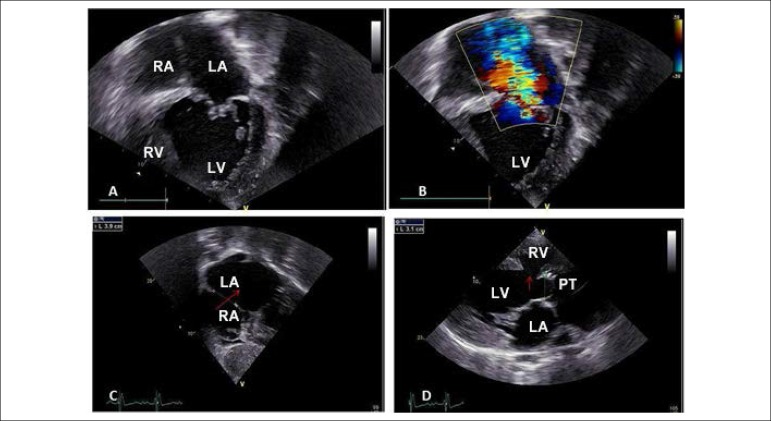
Echocardiogram highlights the marked increase in left heart cavities with right atrioventricular valve atresia and very hypoplastic right ventricle in subcostal view in A; marked mitral regurgitation in B; the large interatrial septal defect (arrow) in subcostal view in C; and the long-axis view image in D showing the interventricular septal defect (arrow) and the pulmonary valve-LV connection, characterizing type IIB tricuspid atresia. RA: right atrium; LA: left atrium; PT: pulmonary trunk; RV: right ventricle; LV: left ventricle.

**Echocardiogram:** Absence of atrioventricular connection on the right, with ventricular-arterial discordance and extensive septal defects, both interatrial (34 mm) and interventricular (22 mm), and posterior deviation of the infundibular septum with pulmonary subvalvular stenosis. Pulmonary trunk dilatation was observed, and the mitral valve showed double dysfunction. The left ventricle (LV) was dilated, with an ejection fraction of 54%. Maximum pressure gradient LV-PT = 58 to 77 mmHg. (Figure 2).

**Magnetic Resonance Image (MRI):** Same findings observed in the echocardiogram.

**Cardiac catheterization:** RV = LV: 110 mmHg; PT: 48 mmHg; PVR: 1.3 UW and SVR: 35.9 UW and Qp/Qs: 5.3/ L.

**Laboratory findings:** Hg: 19.3, Hct: 59%, uric acid: 9.5.

**Clinical Diagnosis:** Type II B tricuspid atresia with extensive septal defects and moderate infundibulum-pulmonary valve stenosis, mitral insuficiency, maintaining pulmonary hyperflow and high arterial saturation, undergoing natural evolution until adulthood.

**Clinical Reasoning:** There were clinical elements leading to a diagnosis of cyanogenic congenital heart disease with marked clinical repercussion, with pulmonary hyperflow. Tricuspid atresia or double LV inflow tract with mild to moderate pulmonary stenosis due to limitation of pulmonary flow, in the presence of auscultation characteristic of associated pulmonary stenosis. The electrocardiogram emphasized LV overload, compatible with the above diagnoses. Echocardiogram and MRI highlighted the diagnostic elements of the defect.

**Differential Diagnosis:** Other cyanogenic heart diseases with pulmonary hyperflow should be recalled with the same pathophysiological picture. Among them, left atrioventricular valve atresia in the presence of a well-developed LV and any other heart disease accompanied by right ventricular hypoplasia.

**Clinical Conduct:** Taking into account the harmonized pulmonary and systemic flows over time, with no signs of hypoxemia and / or heart failure and in the presence of good physical tolerance, the clinical expectant management was considered.

**Comments:** It is known that the different types of tricuspid atresia, whether with pulmonary flow limitation or not, has an unfavorable evolution, with signs of hypoxia or heart failure as early as in the first days of life, which progressively worsens over the first months, until the end of the first year of life. Therefore, the need for surgical intervention in this period. It can be affirmed that cases with tricuspid atresia and a mild repercussion who remain asymptomatic until adulthood are rarely identified.^1^ In this circumstance, they may not require early surgical intervention. Thus, it is important to emphasize that these patients require a stringent and thorough evaluation, in order to be able to determine the most correct conduct for the infant, whether expectant or surgical intervention. This decision becomes even more difficult in adulthood, since heart failure that is observed at a later period, with myocardial dilatation and hypertrophy, and even with cardiac function preservation, is a parameter for an indefinite conduct, given the greater surgical risks in this age group. We did not find reports in the literature that were similar to the case described herein.
